# PD-L1 expression and characterization of its carrier macrophages in placentas with acute and specifically post-SARS-CoV-2 infection

**DOI:** 10.1007/s00418-024-02340-7

**Published:** 2024-11-26

**Authors:** Marina C. Seefried, Johanna Mittelberger, Manuela Franitza, Fabian Garrido, Carl Mathis Wild, Nina Ditsch, Oleksii Protsepko, Christina Kuhn, Christian Dannecker, Peter Altevogt, Udo Jeschke, Marei Sammar

**Affiliations:** 1 Gynecology and Obstetrics, Augsburg University Hospital, Augsburg, Bavaria Germany; 2 DKFZ and University Medical Center Mannheim, Ruprecht-Karl University of Heidelberg, Heidelberg, Germany; 3 Department of Biotechnology Engineering, Braude College of Engineering, Karmiel, Israel; 4 Faculty of Medicine, University of Augsburg, Stenglinstrasse 2, 86156 Augsburg, Germany

**Keywords:** SARS-CoV-2 infection, CD163, PD-L1, Extravillous trophoblast, Macrophages

## Abstract

At the beginning of the coronavirus disease 2019 (COVID-19) pandemic, uncertainties about the virus and its dangers during pregnancy caused great uncertainty and fear, especially among pregnant women. New data suggest an increased risk of obstetric complications, including maternal complications, preterm labor, intrauterine growth restriction, hypertensive disorders, stillbirths, gestational diabetes and risk, of neonatal developmental disorders. In addition, preeclampsia (PE)-like syndromes were also induced by severe COVID-19 infection. Therefore, the aim of this study was to investigate the expression of CD68 and CD163 and PD-L1 on placental tissues from acute covid patients, patients who survived a covid-19 infection and normal term controls that are known to be dysregulated in preeclampsia cases. We examined a total of 60 placentas from women that had given birth to female or male offspring in the University Hospital Augsburg. We investigated ten acute COVID-19 females, ten acute COVID-19 males, ten post-COVID-19 females, ten post-COVID-19 males, ten female term controls, and ten male term controls. Immunohistochemical staining against CD68, CD163, and PD-L1 was performed and the expression of the markers was evaluated with an immunoreactive score (percentage score). Identity of CD163- or PD-L1 expressing cells was analyzed by double immune fluorescence analyses. In opposite to PE, CD163 positive maternal macrophages are significantly upregulated in the decidua of male acute COVID-19 placentas. PD-L1 is significantly upregulated on male acute- and post-COVID-19 decidual immune cells and on male post-COVID-19 extravillous trophoblast cells. Surprisingly the observed effects are related to the fetal gender as they were not observed in female offsprings. Further investigation is necessary to analyze especially the imprinting effect of this infection.

**Supplementary Information:**

The online version contains supplementary material available at 10.1007/s00418-024-02340-7.

## Introduction

At the beginning of the coronavirus disease 2019 (COVID-19) pandemic, uncertainties about the virus and its dangers during pregnancy caused great uncertainty and fear, especially among pregnant women (Meister et al. 2023). In a number of studies this elevated fear was confirmed (Hagenbeck et al. 2020, Schaal et al. 2022, Hagenbeck et al. 2023, Schaal et al. 2023). Since pregnancy is accompanied with a general immunomodulation of the pregnant women, but not necessarily an immune-compromised state, immune changes subject pregnant women to increased susceptibility to viral infection. During the COVID-19 pandemic, pregnant women were more susceptible to serious illness (Accurti et al. 2022). Also, other studies revealed an elevated mortality of pregnant women with COVID-19 infection (Accurti et al. 2022, Ahmad et al. 2022; Ambedkar et al. 2023). New data suggest an increased risk of obstetric complications, including maternal complications, preterm labor, intrauterine growth restriction, hypertensive disorders, stillbirths, gestational diabetes, and risk of neonatal developmental disorders. Overall, there are still controversial concerns about the potential risk for vertical transmission from the fetus to the developing child (Tosto, Meyyazhagan et al. 2023).

Recent studies have revealed that pregnant women who contract severe acute respiratory syndrome coronvirus 2 (SARS-CoV-2) can experience placental dysfunction similar to “preeclampsia-like syndrome”. Whole transcriptome profiling of placental pathobiology in SARS-CoV-2 pregnancies by Stylianou et al. identified placental dysfunction signatures. The study shows that the transcriptional signature of the placenta from acute third trimester pregnancies with SARS-CoV-2 adopts a transcriptional profile aligning with placental dysfunction that was observed in pregnant participants who develop “preeclampsia-like” syndrome (Stylianou et al. 2024). In this context, several studies showed a down regulation of M2 cells in preeclampsia (Yang et al. 2023).

In the absence of vertical transmission to the neonate, maternal SARS-CoV-2 infection altered the transcriptional and functional state in fetal immune cells in circulation and in the placenta. For comparison, spatial proteomic profiling study by Gabby, et al. revealed that the expression of PD-L1 was increased in villitis of unknown etiology, rather than in cases of infectious villitis (Gabby et al. 2024). In the present study, we did not focus on villitis-driven PD-L1 changes but on PD-L1 expression differences in general. Therefore, similarities between SARS-CoV-2 post infection and PE-like syndrome in PD-L1 and its receptor PD1 expression were not described so far. Recent studies showed that pregnant women infected by SARS-CoV-19 exhibit placental dysfunction-increased vasculopathy and inflammation- known as “preeclampsia-like syndrome” (Leavitt et al. 2021; Naeh et al. 2022; Serrano et al. 2023). Our own recent study on PD-L1 expression in the placenta associated with preeclampsia showed, that this immune checkpoint molecule is expressed on decidual macrophages that are also positive for CD163 (representing the M2 subtype) and CD68 (representing a general macrophage marker) (Mittelberger et al. 2023). Therefore, this macrophage marker combination was chosen to identify PD-L1 positive cells within the present study.

In addition, it was reported that there exists persistence of residual COVID-19 virus and immune responses in the placentas of pregnant patients recovered from COVID-19 infection (Wu et al. 2021a, b). The same study showed that infiltration of CD14^+^ macrophages into the placental villi of the COVID-19 recovered pregnant women was higher than that in normal control pregnancies (Wu et al. 2021a, b). Moreover, another study described a distinct subcellular localization pattern of viral RNA in alveolar type 2 cells and alveolar macrophages of pregnant women (Acheampong et al. 2021). Juttukonda et al. investigated the decidual immune response following COVID-19 during pregnancy (Juttukonda et al. 2022). Interestingly, they found a small but significant difference in CD14 staining comparing male and female infants in control but not COVID-19 groups (Juttukonda et al. 2022).

Because a systematic investigation of the expression and characterization of PD-L1 carrying macrophages is missing until now, this study aimed to investigate both CD68/CD163 as well as PD-L1 in acute and post-COVID-19 placental tissue with a specific attention to immune cell populations associated with COVID-19 infection.

## Materials and Methods

### Study subjects

This study was approved by the ethics committee of the Ludwig Maximilian University (LMU) Munich, Germany in July 2021. The placental tissue of 60 placentas, 10 acute COVID-19 females, 10 acute COVID-19 males, 10 post-COVID-19 females, 10 post-COVID-19 males, 10 healthy female term controls, and 10 healthy male term controls, who delivered in the University Hospital Augsburg in the years 2020–2022 were obtained and included into the study after written informed consent. The control group was matched to the COVID-19 and post-COVID-19 study group in pregnancy week, fetal sex, and age of the mother ± 5 years. In order to rule out confounders, healthy patients who fulfilled the following criteria were excluded from the study: preeclampsia, hemolysis, elevated liver enzymes and low platelets (HELLP), intrauterine growth restriction, fertility treatment, signs for systemic inflammation in the blood other than COVID-19, and placentation disorders such as placenta accreta/percreta/increta.

### Inclusion and exclusion criteria and COVID-19 diagnostics

#### Inclusion criteria

-Singleton pregnancies

-Birth between 10/2020 and 12/2022 at Augsburg University Hospital

-Maternal age > 18 years

-Consent to participate in the study

-No history of SARS-CoV vaccination (start of the pandemic, no vaccine authorized yet)

#### Exclusion criteria

-Multiple pregnancies, vanishing twin

-COVID-19 status not known at the time of birth

Evidence of an acute COVID-19 infection was provided by means of a positive rapid test or a positive polymerase chain reaction (PCR) at the time of admission to the delivery room. Symptoms ranged from mild cold symptoms to severe symptoms such as pneumonia, fever, and dyspnea. The detection of inflammatory values in the mother’s blood (c-reactive protein [CRP] or leukocytosis) was not considered specific enough for a COVID-19 infection and was therefore not assessed.

The patients in the “post-COVID” group had experienced a COVID-19 infection at some point during pregnancy, but were COVID negative again at the time of birth and had been symptom-free for at least 14 days. The interval between infection and birth was therefore between at least 2 weeks and a maximum of 38 weeks. The cohort of the healthy control group included patients who had not previously experienced a COVID-19 infection, either during pregnancy or outside of it.

#### Placenta collection

The placentas were preserved in buffered formalin immediately after delivery. The samples were then dissected from the central part of the placenta in the institute for pathology in the University Hospital Augsburg, containing decidua and extravillous and villous trophoblasts. After fixation in buffered formalin, the samples were then embedded in paraffin and cut with a sliding microtome to 2–3 μm slices.

The clinical details of the study population are shown in Table 1.

**Table 1:** Clinical details of the study population; first line for the total group, second line for male fetuses, and third line for female fetuses

	Control (*n* = 20)	Acute COVID-19 (*n* = 20)	Post-COVID-19 (*n* = 20)	*p* value
	Male (*n* = 10)	Male (*n* = 10)	Male (*n* = 10)	
	Female (*n* = 10)	Female (*n* = 10)	Female (*n* = 10)	
Age (years) at delivery total	32.26 ± 3.49	31.26 ± 5.31	30.95 ± 4.14	*p* = 0.57
Male	32.00 ± 3.16	30.60 ± 5.76	31.50 ± 3.78	*p* = 0.66
Female	32.50 ± 3.92	31.67 ± 5.07	30.40 ± 4.60	*p* = 0.68
BMI (kg/m^2^) before pregnancy	24.90 ± 4.81	25.43 ± 4.85	25.47 ± 5.18	*p* = 0.82
	25.96 ± 4.48	25.84 ± 4.64	25.75 ± 4.52	*p* = 0.99
	23.94 ± 5.12	24.98 ± 5.31	25.18 ± 6.00	*p* = 0.72
Gravidity	1.79 ± 1.08	2.32 ± 1.00	2.25 ± 1.33	*p* = 0.27
	1.78 ± 0.97	2.00 ± 0.82	2.10 ± 1.37	p = 0.94
	1.80 ± 1.23	2.67 ± 1.12	2.40 ± 1.35	*p* = 0.17
Parity	1.37 ± 0.50	2.05 ± 0.71	2.05 ± 1.28	**p = 0.02**
	1.44 ± 0.53	1.80 ± 0.63	2.10 ± 1.37	*p* = 0.55
	1.30 ± 0.48	2.33 ± 0.71	2.00 ± 1.25	*p* = **0.02**
Gestational age at delivery	39.25 ± 1.92	39.20 ± 2.02	39.25 ± 2.10	*p* = 0.99
	39.40 ± 1.90	39.30 ± 2.11	39.40 ± 1.90	*p* = 1.00
	39.10 ± 2.03	39.10 ± 2.03	39.10 ± 2.38	*p* = 0.98
Birth weight, g	3178.95 ± 506.60	3268.68 ± 404.91	3165.00 ± 627.41	*p* = 0.96
	3343.78 ± 409.12	3279.50 ± 422.84	3375.00 ± 594.61	*p* = 0.49
	3030.60 ± 559.36	3256.67 ± 409.21	2955.00 ± 616.00	*p* = 0.80
APGAR 10 min	9.89 ± 0.32	9.74 ± 0.56	9.95 ± 0.22	*p* = 0.14
	9.78 ± 0.44	9.60 ± 0.70	10.00 ± 0.00	*p* = 0.19
	10.00 ± 0.00	9.89 ± 0.33	9.90 ± 0.32	*p* = 0.33
Umbilical artery pH	7.26 ± 0.11	7.27 ± 0.09	7.28 ± 0.07	p = 0.58
	7.25 ± 0.11	7.25 ± 0.11	7.28 ± 0.07	*p* = 0.59
	7.26 ± 0.12	7.29 ± 0.06	7.28 ± 0.08	*p* = 0.89

We did not observe significant differences according to fetal sex for age ate delivery, body mass index (BMI), birth weight, gestational age, and APGAR. Significant differences according to fetal sex were only found for parity between the tested groups

### Immunohistochemistry

For immunohistochemistry, the paraffin sections had to be deparaffinized with Roticlear® (Carl Roth, Grafrath, Germany) and afterwards bathed in 100% ethanol. To stop the endogenous peroxidase activity, the samples were then incubated in 3% H_2_O_2_ in methanol for 20 min and rehydrated in a descending alcohol gradient to distilled water. In the next step, the slices were put in a high-pressure cooker for 6 min using boiling sodium citrate buffer with pH 6.0 for antigen retrieval or Tris–EDTA buffer pH 9.0 in case of PD-L1 staining.

Subsequently the slices were treated for 5 min with a blocking solution (Reagent 1; ZytoChem Plus HRP Polymer System IgG kit (Mouse/Rabbit) by Zytomed, Berlin, Germany) for saturating electrostatic charges. Then tissue sections were incubated for 16 h at 4 °C with primary antibodies against CD68, CD163 and PD-L1. After washing the slides with phosphate-buffered saline (PBS), the ZytoChem Plus HRP Polymer System IgG kit (Mouse/Rabbit) was applied (Zytomed) and liquid  Diaminobenzidin (DAB+) Substrate Chromogen System (Agilent Technologies, Santa Clara, USA) was used for visualization of the bound primary antibodies. The slices were counterstained with Mayer’s acid hemalum for 2 min and stained blue for 5 min in tap water. In the following step the samples were dehydrated in an ascending series of alcohol, then treated with Roticlear® (Carl Roth) and cover-slipped with RotiMount (Carl Roth). All antibodies which were used in this study are listed in Table 2.

**Table 2:** List of primary and secondary antibodies for immunohistochemistry/immunofluorescence

Antibody	Isotype	Clone	Dilution	Source
Anti-CD68	Rabbit IgG	Monoclonal; clone D4B9C	1:1000	Cell Signaling, Leiden, The Netherlands
Anti-CD163	Mouse IgG1	Monoclonal; clone OTI2G12	1:2000	Abcam, UK
Anti-PD-L1	Rabbit IgG	Monoclonal; clone EPR19759	1:150	Abcam, UK
Anti-CK7	Mouse IgG1, kappa	Monoclonal; clone OV-TL 12/30	1:200	Agilent, USA
Anti-CD163		Goat-anti-mouse IgG, Cy5 or Goat-anti-mouse IgG, Cy3	1:100 1:100	Jackson Immunotech, USA
Anti-CD68		Goat-anti-mouse IgG, Cy5 or Goat-anti-mouse IgG, Cy3	1:100 1:100	Jackson Immunotech, USA
Anti-PD-L1		Goat anti-rabbit-IgG, AF488	1:500	Thermo Fisher
Anti-CK7		Goat-anti-mouse IgG, Cy5	1:100	Jackson Immunotech, USA

For the evaluation of the quantity of antigen-presenting macrophages, Hofbauer and EVT cells, the number of cells was counted in three image sections at a magnification with a 40 × lens. The total number of cells was then calculated by summing the three areas. For the evaluation of the intensity and distribution patterns of the antigen expression in the extravillous trophoblast and the syncytiotrophoblast the a modified semiquantitative score (percentage score) based on the immunoreactive score of Remmele (IRS) (Remmele and Stegner 1987; Remmele and Schicketanz 1993) was used. The percentage score is calculated by the multiplication of the grade of optical staining intensity (0 = none, 1 = weak, 2 = moderate and 3 = strong staining) and the percentage of positive staining cells.

### Immunofluorescence and microscope characteristics

The double immunofluorescence staining allowed us to characterize specific antigens simultaneously. The same formalin-fixed and paraffin-embedded samples were placed in Roticlear® (Carl Roth) for 20 min for deparaffinization. Subsequently, the sections were panned in ethanol in order of descending concentrations (100%, 70%, 50%) and washed in distilled water. Unmasking of antigens was performed by a 5 min heat pretreatment in a pressure cooker with Tris–EDTA buffer, pH 9.0. After washing in distilled water and PBS for 4 min, incubation with immunofluorescence blocking buffer (Cell Signaling, Leiden, The Netherlands) was performed to prevent unspecific staining. The solution was tipped off after 60 min and the primary antibodies listed in Table 2 were applied at 4 °C for 16 h. After a washing step with PBS the bound primary antibodies were detected at room temperature for 30 min by following secondary antibodies (Table 2).

Following a further washing step in PBS sections were covered with True Black for 1 min at 22 °C to quench tissue autofluorescence and subsequently washed again. After drying the slides were cover slipped using DAPI containing fluorescence mounting medium (Vector Laboratories; USA).

Type and manufacturer of the microscope: Keyence BZ-X800 (neu-Isenburg, Germany).

Objective lens specifications: Plan Apochromat 10 × NA 0.45 and 40 × NA 0.95.

Type and manufacturer of camera: BZ-X800 2/3 Inch Monochrome CCD with 2.83 Mega Pixels and 24 bits.

Type of acquisition software used: BZ-X800 Viewer and BZ-X800 Analyzer; Version of 8th of February 2023.

Image acquisition information: Gain + 6 dB, 1/5 s, 1920 × 1440 and 4080 × 3060 (12.5 Moi Pixels).

Fluorescence filters: see Table 3

**Table 3:** Types of filters used for immunofluorescence

Type	DAPI	GFP	TRITC	Cy5
Company	Keyence	Keyence	Keyence	Keyence
Article number	OP-87762	OP-87763	OP-87764	OP-87766
EX	360/40	470/40	545/25	620/60
DM	400	495	565	660
BA	460/50	525/50	605/70	700/75

### Statistics

We used the statistical SPSS version 24 (SPSS Inc., Chicago, IL, USA) for analysis. The differences across groups were calculated by Kruskal–Wallis non-parametric tests (P). Mann–Whitney non-parametric tests were used to compare two groups. Statistical significance was defined when *p* < 0.05.

## Results

### Expression of maternal CD68/CD163 in the decidua

**Fig. 1:**
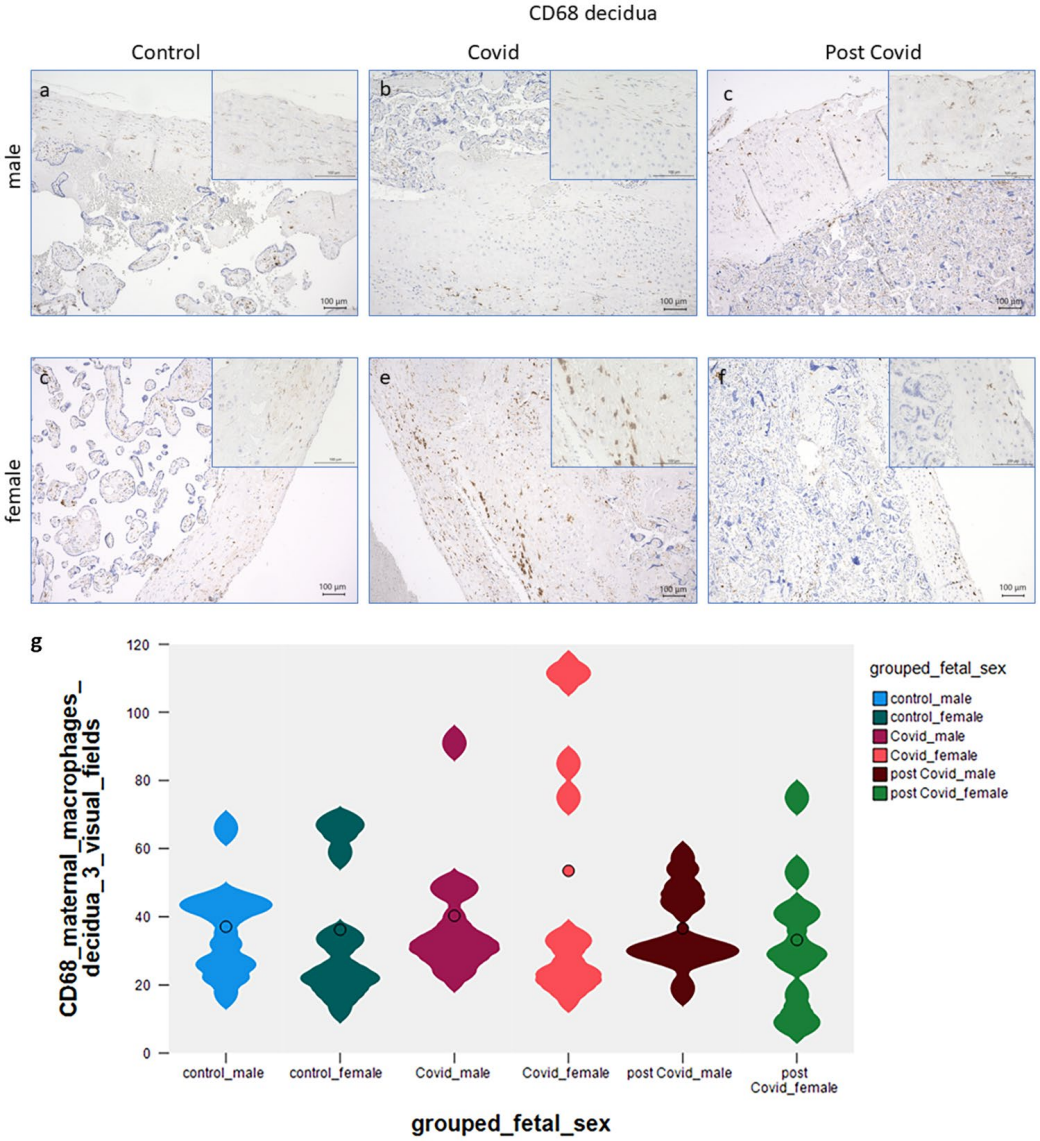
In male decidual control tissue, there was a low number of CD68 positive macrophages (**a**). There was no significant change to male acute COVID-19 (**b**) and male post-COVID-19 cases (**c**). A medium number of CD68-positive macrophages can be found at the fetomaternal interphase in healthy control placentas of female fetuses (**d**), magnification 10 × and 40 × insert. Non-significantly enhanced expression was observed in acute female COVID-19 EVT (**e**), magnification 10× and 40× insert. There is no difference in female post-COVID-19 placentas (**f**) compared with controls. A summary of the staining results is shown as violin plot in (**g)**

Analyses of CD68 in the decidua revealed that there are few CD68 positive macrophages in male control (Fig. 1a) and covid-19 deciduas (Fig. 1b). The number of positive macrophages is non-significantly enhanced in the acute COVID-19 decidua of female fetuses (Fig. 1d control, Fig. 1e female COVID-19 placenta). Post-COVID-19 placentas showed few CD68 positive macrophages in male (Fig. 1c) and female deciduas (Fig. 1f). In all these cases, CD68 is unchanged, a summary is presented in Fig. 1g.

**Fig. 2:**
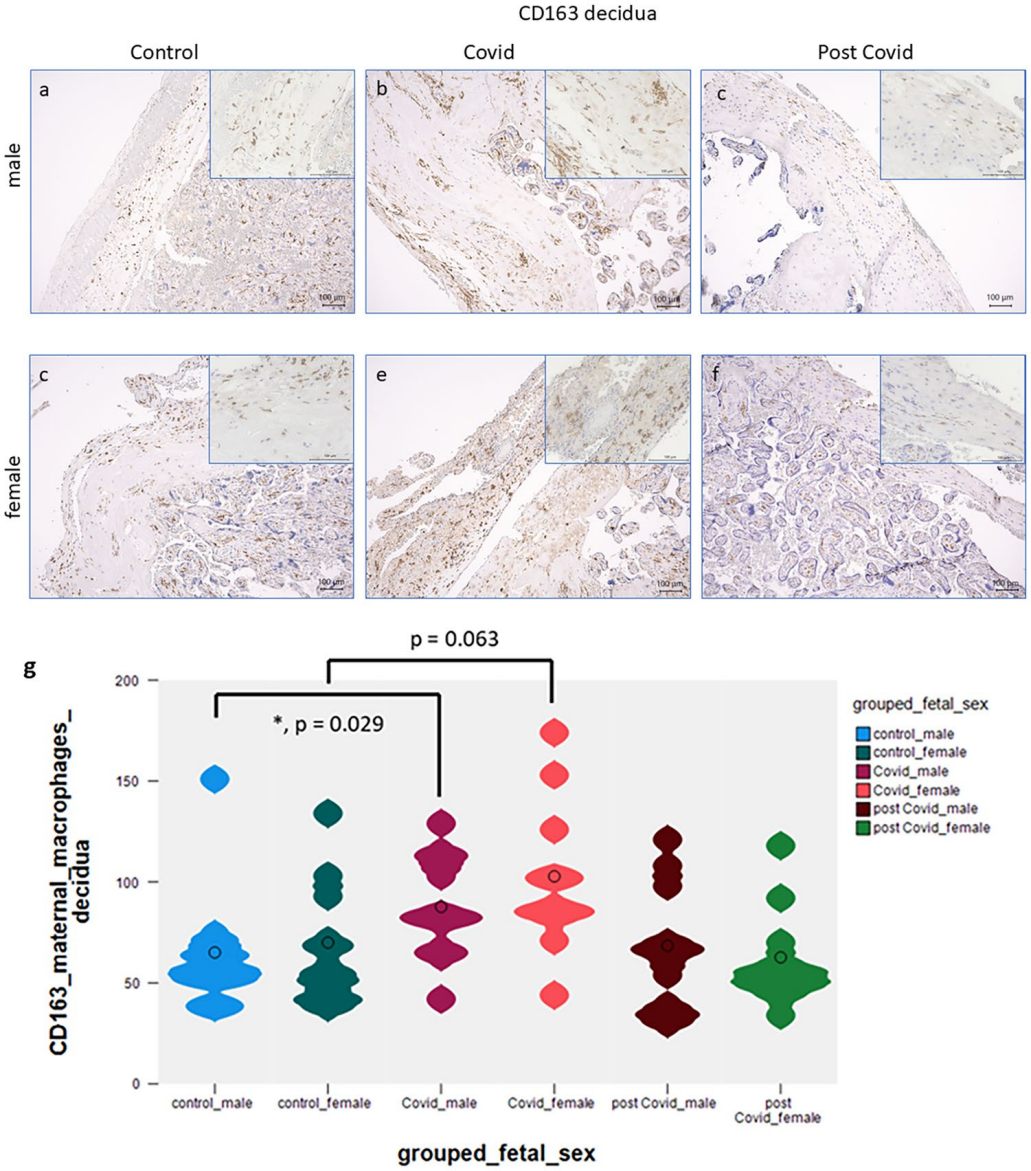
CD163 is expressed in macrophages in low numbers within the fetomaternal interphase in healthy control placentas of male fetuses (**a**), magnification 10× and 40× insert. Significantly enhanced expression of CD163 was observed in acute male COVID-19 macrophages (**b**), magnification 10× and 40× insert. In male post-COVID-19 cases, the expression of CD163 (**c**) is comparable with control cases. CD163 is expressed in moderate numbers of macrophages in healthy control placentas of female fetuses (**d**), magnification 10× and 40× insert. Non-significantly elevated expression of CD163 was observed in female COVID-19 macrophages (**e**), magnification 10× and 40× insert. No differences were observed in female post-COVID-19 cases (**f**). A summary of the staining results is shown as violin plot in (**g**). Significant differences are marked with an asterisk and the *p* value is stated

In contrast, significant differences were observed in CD163 expression in the decidua between male control (Fig. 2a) and male outcome placentas of women with acute COVID-19 infection (Fig. 2b, median control percentage score 8.0 versus median percentage score of 6.5 in acute COVID-19 infection placentas; *p* = 0.029). There was a trend to a significant upregulation of CD163 (*p* = 0.063) between control macrophages (Fig. 2d) and acute COVID-19 macrophages (Fig. 2e) in the decidua of placentas of female origin. There is a low abundance of CD163 in the post-COVID-19 male (Fig. 2c) and female (Fig. 2f) decidua. A summary of all staining results in the extravillous trophoblast (EVT) is presented in Fig. 2g.

### Expression of PD-L1 in the decidua

**Fig. 3:**
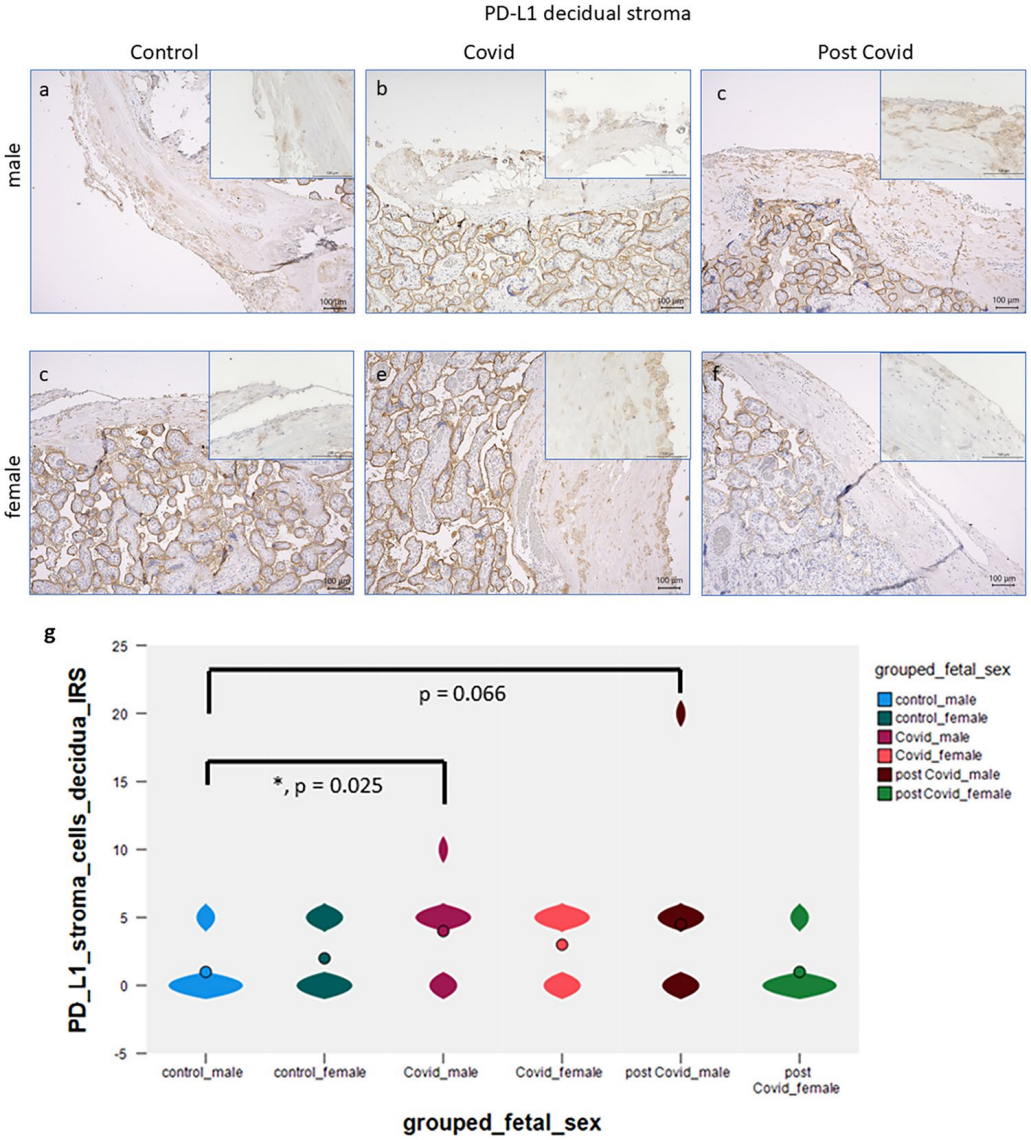
PD-L1 is expressed in the decidual stroma in low numbers in healthy control placentas of male origin (**a**), magnification 10 × and 40x. Significantly enhanced expression of PD-L1 was observed in acute COVID-19 male decidual cells (**b**), and a trend to a significant upregulation was seen in post-COVID-19 male decidual cells (**c**) magnification 10× and insert 40×. In the female placenta, there is no significant difference in stromal PD-L1 expression between controls (**d**), COVID-19 cases (**e**) and post-COVID-19 cases (**f**). A summary of the staining results is shown as violin plot in **g**. Significant differences are marked with an asterisk and the *p* value is stated

PD-L1 is expressed in cells within the decidual stroma of normal control third trimester placentas. Significant differences were observed in PD-L1 expression in cells within decidual tissue between male control (Fig. 3a) and male outcome placentas of women with acute COVID-19 infection (Fig. 3b), median control percentage score 1 versus median percentage score of 4.5 in acute COVID-19 infection placentas; *p* = 0.025. In male post-COVID-19 placentas, there was a trend for an upregulation of PD-L1 expression in decidual cells of post-COVID-19 placentas (Fig. 3c, median percentage score ~ 5; *p* = 0.066) compared with control cells (Fig. 3a, percentage score = 1). In the female placenta, there is no significant difference in stromal PD-L1 expression between controls (Fig. 3d), COVID-19 cases (Fig. 3e) and post-COVID-19 cases (Fig. 3f). A summary of all staining results in decidual cells is presented in Fig. 3g.

### Expression of PD-L1 in extravillous trophoblast cells (EVT)

**Fig. 4:**
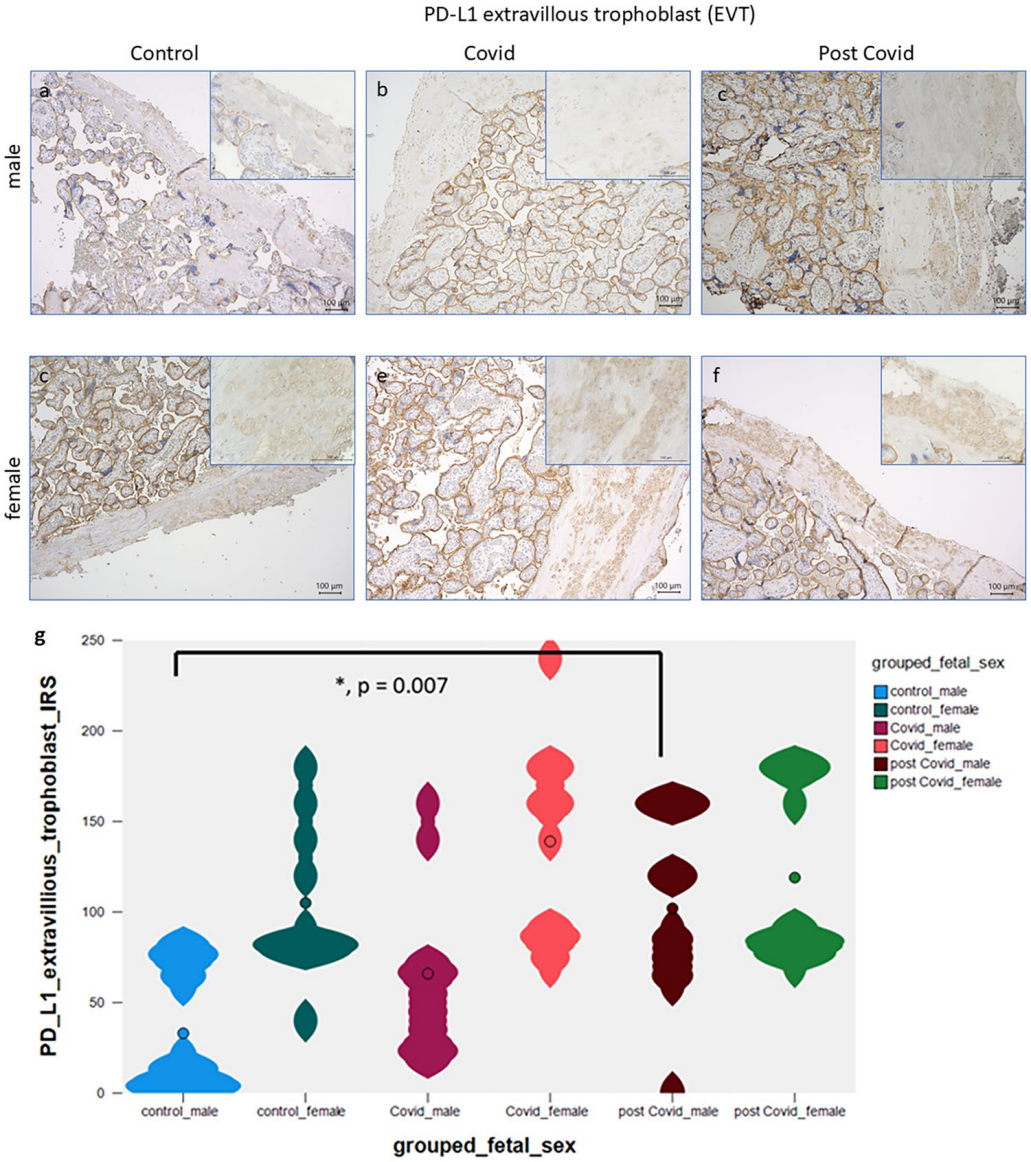
A low or nearly absent expression of PD-L1 was identified in extravillous trophoblast cells (EVTs) in healthy control placentas of male fetuses (**a**), magnification 10× and insert 40×. A significant upregulation of PD-L1 was observed in male post-covid-19 EVTs (**c**), magnification 10× and 40× insert. In male acute COVID-19 cases (**b**), there was no significant difference to controls (**a**). In the female placenta, there was no significant difference in EVT-PD-L1 expression between controls (**d**), COVID-19 cases (**e**), and post-COVID-19 cases (**f**). A summary of the staining results is shown as violin plot in (**g)**. Significant differences are marked with an asterisk and the *p* value is stated

PD-L1 is expressed on a very low level on the extravillous trophoblast cells (EVT) within decidual tissue of normal control third trimester placentas. Significant differences were observed in PD-L1 expression in EVTs between male control (Fig. 4a) and male post-COVID-19 infection EVTs (Fig. 4c; median control percentage score 3.0 versus median percentage score of 11.0 in post-COVID-19 infection placentas; *p* = 0.007). There was no significant change in acute COVID-19 male placentas (Fig. 4b). In the female placenta, there was no significant difference in EVT-PD-L1 expression between controls (Fig. 4d), COVID-19 cases (Fig. 4e) and post-COVID-19 cases (Fig. 4f). A summary of all staining results in EVTs is presented in Fig. 4g.

### Identification of PD-L1 expressing cells within the decidua

**Fig. 5:**
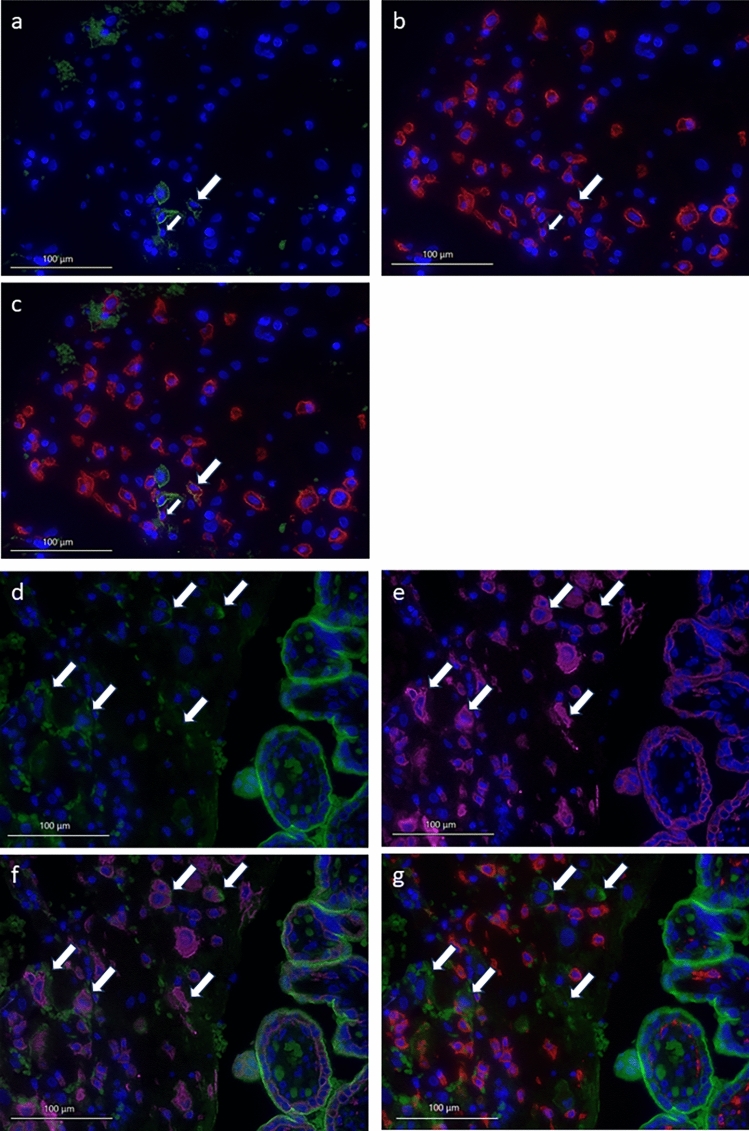
PD-L1-positive cells (green fluorescence) of male acute covid-19 decidual cells marked with white arrows (**a**) are in part also positive for CD163 (red fluorescence) (**b**). PD-L1 positive cells are again marked by white arrows; magnification 40×. Triple staining of PD-L1 (green), CD163 (red), and DAPI (blue) is shown in (**c**) (40× magnification). PD-L1-positive cells (green fluorescence) of post-COVID-19 placentas with male fetuses (**d**) are marked in the decidua with white arrows. PD-L1 positive cells of the syncytiotrophoblast are easily recognizable on the right side of the picture. CK7 staining of the white arrow marked PD-L1-positive cells (pink fluorescence) (**e**) revealed proof of the origin of these cells as extravillous trophoblast cells (EVTs). Triple staining of PD-L1 (green), CK7 (pink), and DAPI (blue) is shown in **f** (40× magnification). CD163-positve macrophages (red fluorescence; **g**) seems to be closely connected to PD-L1 positive EVTs that are again marked with white arrows

Within the male COVID-19 decidua, there was an abundance of PD-L1-positive cells stained in green (Fig. 5a). CD163 (red fluorescence) was used as M2 macrophage specific marker (Fig. 5b). Double expression of PD-L1 and CD163 identified PD-L1-positive cells at least in part as macrophages (Fig. 5c) marked with white arrows.

In post-COVID-19 male placentas, there are different cells that express PD-L1 (Fig. 5d) marked with white arrows. Cytokeratin 7 (CK7) was used as a marker for extravillous trophoblast cells (EVT, Fig. 5e, pink fluorescence). Double immune fluorescence staining showed that PD-L1-positive cells, marked with white arrows, do also express CK7 (Fig. 5f) and therefore are EVT in post-COVID-19 placentas.

In addition, double immune fluorescence staining for CD163 (red fluorescence) and PD-L1 (green fluorescence, marked by white arrows; Fig. 5g) showed that maternal M2 polarized macrophages are localized in close vicinity to PD-L1 positive EVTs in post-COVID-19 placentas.

## Discussion

Within this study, we could show that there is an infiltration of maternal CD68-positive macrophages and CD163-positive M2-polarized macrophages in the SARS-CoV-2-infected placenta. No significant differences were detected in CD68-positive cells in infected placentas compared with control. In the contrary, CD163-positive macrophages are significantly upregulated in male COVID-19 placentas, whereas there is only a trend for such upregulation in female placentas from infected placentas compared with the uninfected negative controls. In addition, the immune checkpoint molecule PD-L1 is also upregulated in the male acute covid-19 and post-covid-19 decidua. Double immune fluorescence staining showed that these PD-L1 positive cells are CD163-positive M2 macrophages on one hand and in addition, extravillous trophoblast cells on the other hand.

A massive infiltration of M2-activated macrophages was observed in the decidua from male of post-infected pregnancies which reflects the part of inflammatory responses in the placenta upon SARS-CoV-2 infection. These results are in agreement with work from Lissenya B. 2024, analyzing placentas from women who have been tested positive for SARS-CoV-2 at the time of delivery. The results show migration of CD163-positive monocytes/macrophages into the intervillous space as well as increased numbers of Hofbauer cells (HBC) within the chorionic fetal villi compared with the uninfected negative controls. The authors reported a significantly higher number of CD163-positive HBC in the chorionic plate (CP) and a slight increase in the number of macrophages in the maternal decidua. Moreover, transcriptome analysis of placental tissue and of acutely infected placental cell clusters revealed the increased expression chemokines, cytokines, and inflammatory-related genes (Argueta et al. 2022).

Here we analyzed placentas from women who tested positive for SARS-CoV-2 at the time of delivery to determine the extent of infection and impact on the inflammatory state of the placental tissues. PD-L1 is a transmembrane protein that is strongly involved in immune modulation, serving as checkpoint regulator. Interaction with its receptor PD-1 induces an immune-suppressive signal, which modulates the activity of T cells and other effector cells involved in maintenance of maternal antifetal tolerance (Beenen et al. 2022, Mittelberger et al. 2022). Although we expected that at the postinfection time the immune cells will return to the normal levels, our results show an activated status of the M2 macrophages, which were expressing PD-L1 at post infection (at delivery). This observation could be explained by the capacity of the immune cells to be educated and remain alerted and ready to combat future infections.

Our results revealed upregulation of the number of CD163 positive M2 macrophages in the decidua of male postinfected SARS-CoV-2 infection. This upregulation and activation of M2 macrophages was associated with upregulation of the PD-L1 receptor on M2 macrophages. In the contrary PD-L1 is downregulated in preeclamptic male placentas (Mittelberger et al. 2023), whereas it is upregulated in the present study in the decidua of SARS-CoV-2 infected male placentas. Taking into consideration the complexity of the preeclampsia, the PD-L1- and CD163-positive macrophages might play a distinct role in SARS-CoV-2 infection placenta compared with PE.

Another striking finding was that we identified PD-L1 positive maternal macrophages (CD163-positve) in the acute COVID-19 male decidua; this upregulation (although not significant) was also found in the post-COVID-19 situation but only in male placentas. There are also other studies, that found sex-specific differences in COVID-19 placentas (Wu et al. 2021a, b). Bordt et al. characterized placental immune responses in women who were infected with SARS-CoV-2 during pregnancy (Bordt et al. 2021). The study revealed differential placental immune responses between male and female fetuses, which were associated with decreased antibody transfer to male fetuses (2021, Bordt et al. 2021). Another very recent study by Edlow et al. showed that maternal SARS-CoV-2 positivity was associated with a statistically significant elevation in risk for neurodevelopmental diagnoses at 12 months among male offspring (Edlow et al. 2023). Therefore, male offspring seems to be specifically endangered after a SARS-CoV-2 pregnancy.

The time of infection in pregnancy is a crucial point to consider for the analyses of SARS-CoV-2 effects. Gene expression analysis by Enninga et al**.** showed a significant indirect impact of SARS-CoV-2 infection on gene expression profiles in inner mesenchymal HBCs, with limited effect on lining CTB cells isolated from pregnant subjects during the three trimesters of pregnancy (Enninga et al. 2024). The gene expression profiles in both cell types were dependent on the time of infection during gestation (Enninga et al. 2024). Based on our knowledge, it is the percentage score study that shows the expression of PDL1 in EVT at the fetal maternal interface and their close contact (close proximity) with the maternal immune cells. We speculate that the M2 activation (CD163 positive) might be initiated by PDL1 interaction with soluble PD1 receptors released from immune effector lymphocytes.

Further studies on larger cohorts of patients are required to confirm the outcome of this study.

Our study showed differences in PD-L1 in the decidua. Double immunofluorescence showed that PD-L1 positive cells are to a major extend extravillous trophoblast cells at least in the male post-COVID-19 placenta. There we also found a close interaction with maternal CD163-positive M2-polarized macrophages. This interaction could stand for a more immunosuppressive decidual environment in the post-COVID-19 situation.

An interplay between viral infection, pregnancy-specific immune shift and endothelial dysfunction may lead to negative pregnancy outcomes (Celewicz et al. 2023). However, mechanisms through which SARS-CoV-2 infection predisposes pregnancies to these preeclampsia-like pathological features are largely unclear.

## Conclusion

The data of our study showed an upregulation of CD163 positive maternal macrophages and a higher PD-L1 expression in acute and post-COVID-19 male placentas. In addition, we identified a significant upregulation of PD-L1 in post-COVID-19 male extravillous trophoblast cells. These post-COVID-19 placental changes could to some degree affect at least the male post-COVID-19 offspring. Additional studies are required to confirm these effects.

## Supplementary Information

Below is the link to the electronic supplementary material.

Supplementary file 1 (PDF 125 KB)

## Data Availability

No datasets were generated or analyzed during the current study.
