# Supplementary material for: PD-L1 expression and characterization of its carrier macrophages in placentas with acute and specifically post-SARS-CoV-2 infection
Source: Histochem Cell Biol. 2024 Nov 26;163(1):9. doi: 10.1007/s00418-024-02340-7 (PMC11599338; doi:10.1007/s00418-024-02340-7)
Supplement: Supplementary file 1 — (PDF 125 KB) [file 418_2024_2340_MOESM1_ESM.pdf]

77	151	0	300	0		1	
123	63	40	300	10		3	
72	108	60	300	0		5	
72	53	10	180	0		1	
174	83	20	180	0		3	
72	39	120	300	20		5	
95	39	10	300	0		1	
67	42	30	160	0		3	
92	63	120	270	5		5	
112	63	0	160	0		1	
109	67	20	160	5		3	
64	70	160	270	5		5	
78	60	0	160	0		1	
75	103	50	270	0		3	
50	30	0	160	0		5	
106	52	70	160	5		1	
126	129	70	180	5		3	
94	67	80	270	0		5	
76	74	20	160	0		1	
59	115	60	180	5		3	
66	34	160	270	5		5	
86	38	60	70	0		1	
40	80	70	180	5		3	
58	54	70	160	0		5	
69	53	80	270	5		1	
103	84	160	300	5		3	
105	121	90	180	5		5	
83	68	80	180	0		1	
106	111	140	180	5		3	
101	98	160	180	5		5	
55	71	40	270	5		2	
101	102	80	300	0		4	
47	92	80	180	0		6	
79	103	140	300	5		2	
73	71	70	300	5		4	
61	53	160	300	5		6	
97	38	80	180	0		2	
85	83	180	300	5		4	
74	34	180	300	0		6	
70	46	180	300	5		2	
100	44	90	160	0		4	
89	45	180	300	0		6	
59	53	160	300	5		2	
126	102	180	300	5		4	
83	52	90	300	0		6	
101	66	120	180	0		2	
97	85	160	300	5		4	
50	47	180	300	5		6	
42	55	80	270	0		2	
111	88	240	300	5		4	
52	118	80	300	0		6	
89	93	80	180	0		2	
71	174	160	80	5		4	
76	60	70	180	0		6	
56	41	90	270	0		2	
60	126	90	160	0		4	
78	56	90	300	0		6	
57	134	80	180	0		2	
108	153	140	270	0		4	
111	70	80	160	0		6	
CD68	CD163	PD-L1 EVT	PD-L1 Syncytiotrophoblast	PD-L1 decidua		cases	
						1	control male
						2	control female
						3	Cov-19 male
						4	Cov-19 female
						5	post Cov male
						6	post Cov female
